# Beware of Rattling Underfoot: Cystic Artery Pseudoaneurysm in Acute Cholecystitis With a Cholecystostomy

**DOI:** 10.7759/cureus.33564

**Published:** 2023-01-09

**Authors:** Umasankar Mathuram Thiyagarajan, Amirtha Ponnuswamy, Andrew Hindmarsh

**Affiliations:** 1 Transplantation and Hepatobiliary-Pancreactic Surgery, University of Alberta Hospital, Edmonton, CAN; 2 Family Medicine, Epsom and St Helier Hospital NHS Trust, London, GBR; 3 General and Oesophagogastric Surgery, Addenbrooke's Hospital, Cambridge University Hospitals NHS Foundation Trust, Cambridge, GBR

**Keywords:** gastrointestinal bleeding, cholecystostomy, pseudoaneurysm, gallstones, cholecystitis

## Abstract

Cystic artery pseudoaneurysm (CAP) is a very rare complication of acute cholecystitis. The pathogenesis of CAP in the context of cholecystitis is unknown but is possibly related to the inflammatory process in the vicinity of the cystic artery, leading to weakness in the wall of the artery.

Though CAP has been reported in the literature, our patient had a unique presentation in the presence of a cholecystostomy catheter in situ. There were no risk factors for CAP in our patient including usage of anticoagulants, trauma, or surgical procedures. Fortunately, the blood-stained fluid in the cholecystostomy catheter effluent alerted the clinical team to a possible vascular complication in the background of ongoing cholecystitis. This finding should serve as a warning sign to alert clinicians to the possibility of CAP-beware of rattling underfoot.

## Introduction

Cystic artery pseudoaneurysms (CAP) are rare and have been reported to occur as a complication of acute cholecystitis when CAP formation is likely to result from damage to the vessel wall caused by the inflammatory process [1]. There are also reports of CAP following cholecystectomy, where traumatic injury of the cystic artery leads to the subsequent development of CAP [2]. CAP can present as haemobilia, jaundice, right upper abdominal pain, and fever [3].

Gallstone disease is a common and significant health problem in Western countries. The prevalence of gallstones is about 10-15% in the adult population [4]. Approximately 20% of patients with gallstones develop symptoms including biliary colic, cholecystitis, and gallstone pancreatitis [5]. The cost of managing gallstone-related diseases is estimated to be 6.5 billion dollars per annum in the United States where 700,000 cholecystectomies are performed each year [4]. Around 70,000 cholecystectomies are performed for symptomatic gallstones in the United Kingdom per year [5].

Although gallstone disease is common, CAP is a rare complication with only a few cases reported in the medical literature. Our patient underwent cholecystostomy to treat his complicated acute cholecystitis and developed CAP within a short span of time following that intervention, which is extremely rare.

## Case presentation

A 70-year-old Caucasian man presented to the emergency department with a 24-hour history of epigastric/right upper abdominal pain and fever. He had been experiencing vague intermittent upper abdominal discomfort and nausea for three weeks prior to this presentation. On examination, he was tachycardic (pulse rate 100/minute), normotensive (110/80 mm Hg), febrile (temperature 38 degrees Celsius), and clinically jaundiced. His abdomen was soft with tenderness elicited in the epigastrium and right hypochondrium.

He had a past medical history of reflux oesophagitis and hypothyroidism for which he was taking lansoprazole and thyroxine respectively and a family history of ischaemic heart disease.

Investigation

Preliminary blood investigations including the biochemical profile and complete full blood count are listed in Table 1 below. Urinalysis and coronavirus disease (COVID-19) screenings were negative.

**Table 1:** Clinical laboratory reference values

Parameter	Patient’s value	Reference Values
White cell count (WCC)	12 x 10^9^ /L	3.5 - 10.5 × 10^9^/L
Bilirubin	79 µmol/litre	3 - 17 µmol/litre
Alanine aminotransferase (ALT)	95 IU/litre	17- 63 IU/litre
Aspartate aminotransferase (AST)	71 IU/litre	15 - 34 IU/litre
Alkaline phosphatase (ALP)	98 IU/litre	40 - 120 IU/litre
International normalised ratio (INR)	1.5	0.9 - 1.2
Amylase	45 IU/litre	25 - 115 IU/litre
C-reactive protein (CRP)	114 mg/L	≤ 10 mg/litre

Chest and abdominal X-rays were normal, and an urgent abdominal computed tomography (CT scan) was performed which demonstrated acute cholecystitis with associated pericholecystic fluid. There was no evidence of gallbladder perforation or adjacent hepatic abscess. The patient was jaundiced with deranged liver function tests. Magnetic resonance cholangiopancreatography (MRCP) was also performed which showed acute calculous cholecystitis with no bile duct stones.

Treatment

The patient was admitted as an inpatient, received analgesia, and was started on intravenous amoxicillin/clavulanic acid therapy. Despite treatment with appropriate antibiotics he continued to have intermittent fevers with a rising CRP, the amoxicillin/clavulanic acid (1.2 grams intravenous every eight hours) was switched on Day 2 to piperacillin with tazobactam (4.5 grams intravenously every six hours) for better microbiological cover. A further CT scan of his abdomen was performed seven days following admission which demonstrated worsening inflammatory stranding around the gallbladder and porta hepatis with biliary dilatation. We, therefore, proceeded with a cholecystostomy to decompress the gallbladder. A percutaneous transperitoneal cholecystostomy catheter was placed in the gallbladder under ultrasound guidance and was left on free drainage. The procedure was uncomplicated. Following the insertion of the cholecystostomy catheter, the patient’s clinical condition slowly improved, with an associated decrease in his WCC and CRP. A subsequent cholecystogram showed good positioning of the cholecystostomy catheter within the gallbladder and free flow of contrast to the duodenum.

**Figure 1:**
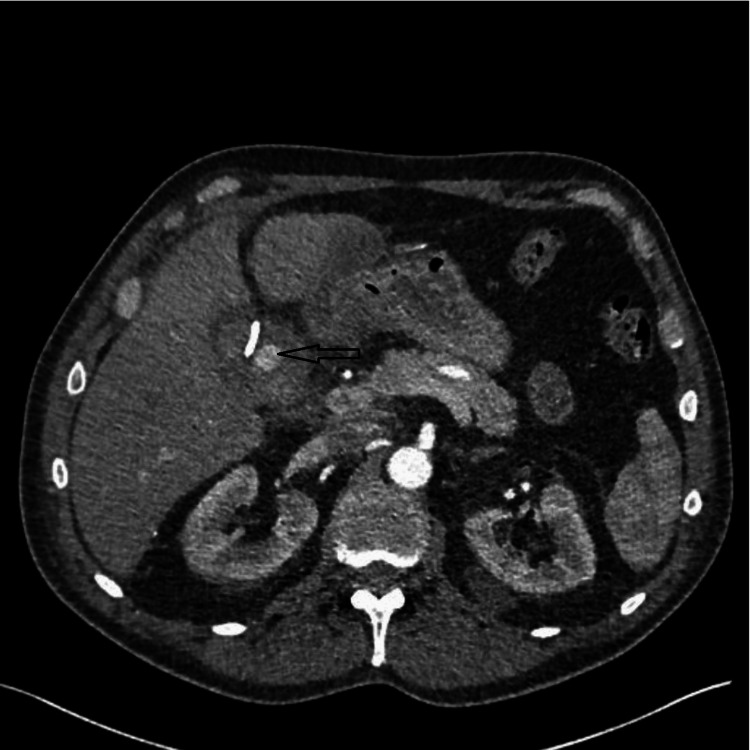
CT angiogram showing a pseudoaneurysm of the cystic artery within the gallbladder (see arrow)

Given the patient’s clinical improvement, a plan was made to discharge him with the cholecystostomy catheter in situ, with an outpatient clinic follow-up in two weeks. However, 10 days after the insertion of the cholecystostomy we noted blood-stained fluid in the cholecystostomy catheter bag. Although the patient was well, with no clinical signs of bleeding (normal pulse rate, blood pressure, and stable haemoglobin), we proceeded with an urgent triple-phase CT scan of his abdomen. The CT scan revealed a 23 x 11 mm pseudo-aneurysm within the gallbladder lumen arising from the cystic artery (Figure 1). The gallbladder lumen was filled with hematoma, and the cholecystostomy catheter remained appropriately placed within the gallbladder.

**Figure 2:**
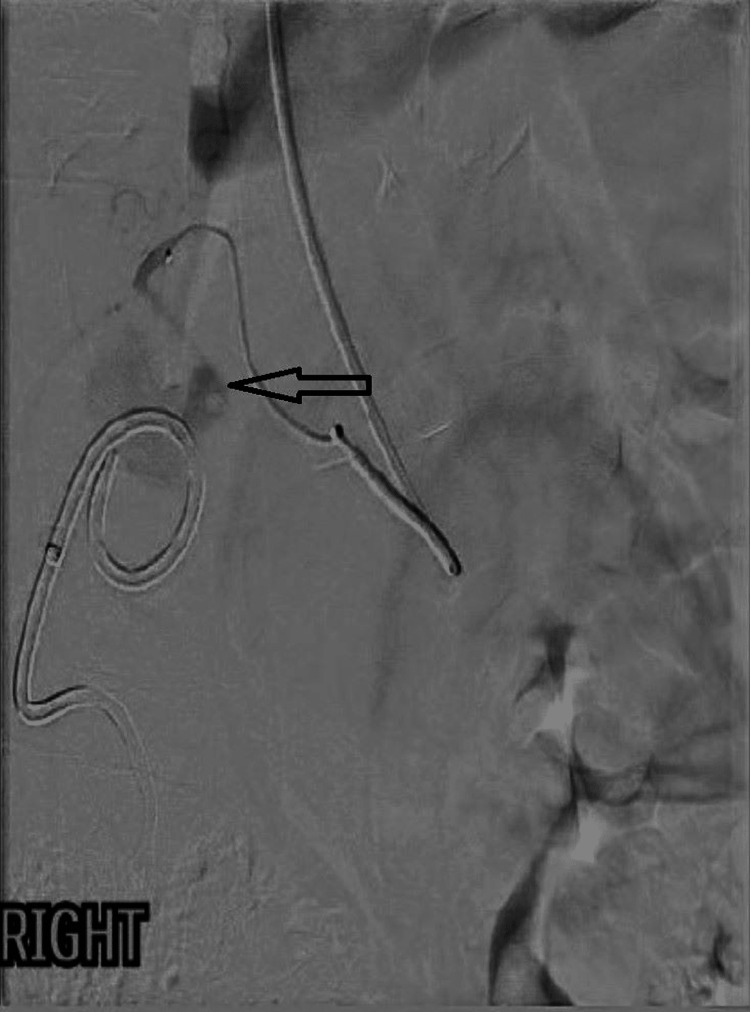
Transcatheter angiogram showing pre-embolisation of the pseudoaneurysm of the cystic artery

**Figure 3:**
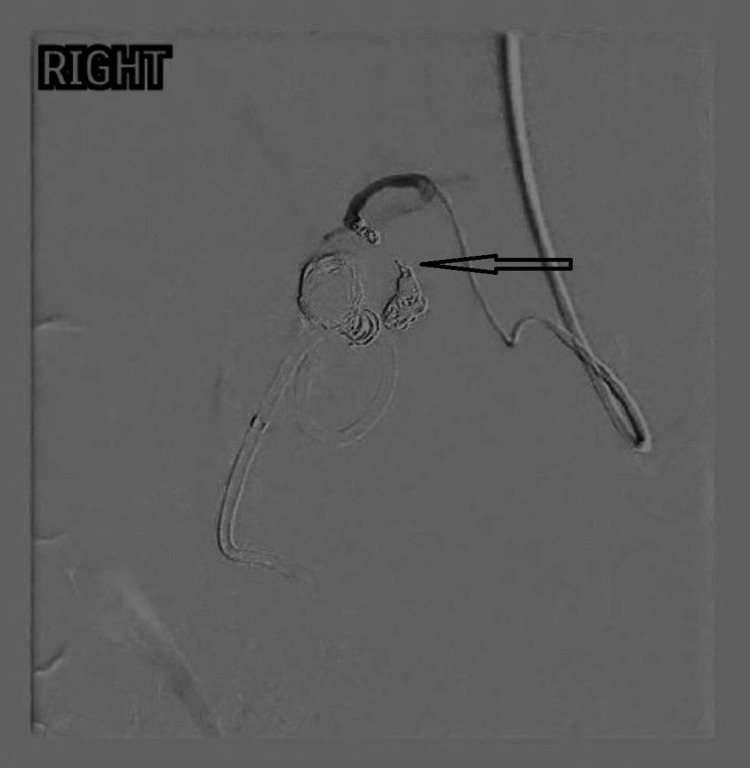
Transcatheter angiogram showing post-embolisation of the pseudoaneurysm of the cystic artery with coils in place

Due to the high risk of significant bleeding from the CAP, urgent consultation with the on-call interventional radiology team was sought. A transcatheter angiogram through the right radial artery showed a bilobed pseudoaneurysm of the cystic artery (Figure 2). The pseudoaneurysm and parent artery were safely embolised with interlock coils with the satisfactory exclusion of pseudoaneurysm from the cystic artery (Figure 3).

Outcome and follow up

The patient was safely discharged a week after transcatheter embolisation of the CAP and attended the clinic with follow-up imaging. A CT scan of his abdomen performed at four and eight weeks following discharge from the hospital showed improving cholecystitis with no further abdominal collection. The patient will be undergoing open cholecystectomy in the coming weeks due to their recent complicated course of cholecystitis with a cholecystostomy in situ.

## Discussion

CAP is a rare but recognised complication of acute cholecystitis, pancreatitis, and post-biliary surgery [1,3,6]. However, CAP in the context of acute cholecystitis and a cholecystostomy catheter is unusual, with only one previous case reported in the literature. In that case, the patient had a number of co-morbidities including end-stage renal failure needing haemodialysis, peripheral vascular disease, and diabetes. They developed acute cholecystitis following a coronary artery bypass graft. As the patient was on dual antiplatelet therapy following their cardiac surgery, cholecystectomy was deferred, and a cholecystostomy catheter was placed to drain their gallbladder. The cholecystostomy catheter had been in situ for four months before CAP was detected [2]. The authors hypothesise that dual antiplatelet therapy contributed to the development of CAP in this case by preventing the thrombosis which would be expected to occur in the cystic vessels in response to inflammation.

In our case, the patient developed a CAP within 10 days of placement of the cholecystostomy catheter. The CAP developed in spite of the patient’s clinical improvement and reduction in inflammatory markers following insertion of the catheter, which indicated resolution of their cholecystitis. In addition, the patients did not receive any antiplatelet agents during their inpatient stay. Fortunately, the blood-stained fluid in the cholecystostomy catheter effluent alerted the clinical team to a possible vascular complication in the background of ongoing cholecystitis.

The cause of the CAP in this case is uncertain. It may be related to the inflammation associated with the patient’s episode of acute cholecystitis. Alternatively, the CAP may have resulted from trauma to the cystic artery during the insertion of the cholecystostomy catheter, or possibly a combination of the two processes.

We feel that this is very unique, rare occurrence from which lessons can be learned. Improving patients’ clinical improvement with normalisation of their inflammatory markers does not preclude the development of a CAP in the presence of a cholecystostomy catheter. Although CAP is a rare complication of acute cholecystitis and insertion of a cholecystostomy catheter, it has the potential to cause massive upper gastrointestinal haemorrhage (haemobilia) which could result in increased mortality and morbidity [7,8].

## Conclusions

Cystic artery pseudoaneurysm (CAP) is a rare but serious complication in patients with acute cholecystitis. Insertion of a cholecystostomy catheter may cause trauma to the cystic artery and increase the likelihood of developing a CAP in the context of acute cholecystitis. Blood-stained cholecystostomy effluent should alert the clinicians on the possibility of a CAP-beware of rattling underfoot. The presence of blood-stained effluent in the cholecystostomy catheter requires an urgent triple-phase CT scan of the liver for diagnosing CAP. Percutaneous transcatheter embolization of the CAP is the treatment of choice with minimal complications.
